# Decidual macrophages derived NO downregulates PD-L1 in trophoblasts leading to decreased Treg cells in recurrent miscarriage

**DOI:** 10.3389/fimmu.2023.1180154

**Published:** 2023-07-14

**Authors:** Yonghong Zhang, Huiyan Wang, Panpan Qiu, Jingwen Jiang, Xuhong Wu, Jie Mei, Haixiang Sun

**Affiliations:** Center for Reproductive Medicine and Obstetrics and Gynecology, Nanjing Drum Tower Hospital, The Affiliated Hospital of Nanjing University Medical School, Nanjing, China

**Keywords:** decidual macrophage, NO, YY1, PD-L1, trophoblast, Treg, recurrent miscarriage

## Abstract

**Introduction:**

Placental trophoblasts contribute to regulatory T (Treg) function *via* the programmed cell death-1 (PD-1)/PD-1 ligand 1 (PD-L1) pathway during normal pregnancy. Decreased expression of PD-L1 in trophoblasts was closely associated with Treg deficiency in the development of pregnancy failure. Thus, targeting PD-L1 might be a novel therapy to prevent pregnancy loss. However, the mechanisms for modulating the expression of PD-L1 in trophoblasts are an enigma.

**Methods:**

The proportion of decidual Treg cells, and the profile of decidual macrophages (DMs) sampled from women with normal pregnancy (NP) and recurrent miscarriage (RM) were evaluated by flow cytometry. The expression of Yin and Yang 1 protein (YY1) and PD-L1 in human villous were measured by Immunohistochemistry (IHC), qRT-PCR and western blot. The determination of soluble PD-L1 (sPD-L1) in serum from NP and RM, and trophoblast conditioned media (TCM) was performed by the PD-L1 SimpleStep ELISA kit. Knockdown of YY1 was processed in the human trophoblast derived cell lines, HTR-8 and Bewo, with siYY1 transfection. Peripheral naïve CD4^+^ T cells were isolated from women with NP for the *in vitro* culture. The percentages of Treg cells differentiated from peripheral naïve CD4^+^ T cells were measured by flow cytometry. The interaction between YY1 and *CD274* was proved by CHIP. The expression of inducible nitric oxide synthase (iNOS) in decidua was evaluated by IHC. The level of NO in serum from women with NP and RM was determined by the Griess reagent system. The effects of NO on YY1 were determined by the *in vitro* culture of HTR-8 cells with the NO donor, SNAP. The *in vivo* model comprising twelve pregnant mice and underwent different treatment. The percentages of Treg cells in murine uterus were measured by flow cytometry. Similarly, Western blot and IHC were performed to determine the expression of YY1 and PD-L1 in murine placenta.

**Results:**

Decreased expression of YY1 and PD-L1 in trophoblasts and lower proportion of decidual Treg cells were observed in patients with RM. Knockdown of YY1 contributes to a lower expression of YY1 and PD-L1. Soluble PD-L1 in the supernatant from HTR-8 cells was also decreased with siYY1 administration. Lower Treg differentiation was observed in the presence of supernatant from HTR-8 cells treated with siYY1. CHIP analysis revealed that endogenous YY1 directly occupied the promoter region of the *CD274* (PD-L1) gene. Accompanied with increased M1 DMs, higher NO was observed in serum sampled from patients with RM. In the presence of Reduced expression of YY1 and PD-L1 was observed in HTR-8 cells with the treatment of SNAP. Furthermore, less Treg differentiation was observed with SNAP treated TCM. Moreover, our *in vivo* data found that YY1 deficiency was associated with decreased PD-L1, which further resulting in less Treg differentiation and Treg deficiency at the maternal-fetal interface and increased embryo loss.

**Discussion:**

Our work found the modulatory capacity of YY1 on PD-L1 in trophoblasts during early pregnancy. Furthermore, reduced YY1 was supposed resulting from higher levels of NO produced from the M1 DMs in RM.

## Introduction

Human pregnancy is generally considered a unique immunologic condition requiring proper modifications of the maternal immune system. This complex process is associated with protecting both the mother and fetus against pathogen invasion, and the maternal tolerance to the semi-allogenic fetus with paternal antigens to promote fetal growth and development (1). Failure to maintain maternal tolerance to the fetus may lead to pregnancy loss, such as recurrent miscarriages (RM), as well as later-onset pregnancy complications resulting from disturbed implantation and placental morphogenesis, such as pre-eclampsia (2–7).

Several mechanisms of maternal tolerance emerge in early pregnancy to control allo-reactive immune responses that may threaten fetal survival. Among the various mechanisms underlying maternal tolerance, regulatory T (Treg) cells are essential for embryo implantation and early placental development (8–10). The lower proportion of Treg cells are closely associated with implantation failure, pregnancy loss, or intrauterine fetal growth restriction (2, 3, 11). Several studies have observed Treg cells in the decidua from early pregnant women, and lower percentages of decidual Treg cells were found in women with RM, indicating the role of Treg cells in the development of RM (12). Therefore, targeting Treg cells by enhancing the Treg cell pool and their suppressive capacity was supposed as an effective method to prevent pregnancy loss. However, our current knowledge on the modulators for Treg cell population at the maternal–fetal interface is still limited.

Placenta-derived trophoblasts mediate the contact between the maternal decidua and blastocyst and play an active role in remodeling the immunologic microenvironment at the implantation site (1). Trophoblast-originated factors are able to recruit and modulate the differentiation and function of immune cells at the maternal–fetal interface throughout pregnancy (13). The former findings showed that Treg differentiation might be modulated by trophoblast *via* the engagement of programmed cell death 1 (PD-1) and its ligand 1 (PD-L1) during pregnancy, whereas PD-L1 supplementation significantly enlarged the Treg cell pool in preeclamptic rats (14). Therefore, targeting PD-L1 is an idle method to promoting Treg immunity. However, the mechanisms modulating the expression of PD-L1 in trophoblasts has not been elucidated.

Decidual macrophages (DMs) are one of the most abundant clusters of immune cells; both M1 and M2 DMs were observed in early pregnancy (15). M1 macrophages are more effective at antigen clearance and promoting T helper-1 (Th1) immune response, whereas M2 macrophages have the immunosuppressive capacities, contributing to Th2 immune response (16, 17). Pregnancy has been proposed as a dynamic and highly regulated immunologic process. Early pregnancy is generally considered a Th1- or Th1/Th2-dominated immune status, thus DMs showing an M1 or M1/M2 mixed profile (18). M1 DMs produce diverse soluble factors, including nitric oxide (NO), and proinflammatory cytokines, such as IL-1β, IL-6, and TNF-α. NO is a water soluble, free-radical gas involved in many biological processes (19, 20). NO is produced by activated M1 macrophages *via* inducible nitric oxide synthase (iNOS). The inhibition of NO on Yin and Yang 1 protein (YY1), which further inhibits PD-L1 expression, has been reported previously (21, 22).

YY1 is a widely distributed transcription factor from the polycomb-group protein family. YY1 participates in the modulation of various biological processes, such as cell survival, embryogenesis, differentiation, proliferation, and metastasis *via* DNA– and protein–protein interactions with numerous partners (23, 24). Tian et al. revealed the modulation of YY1 on matrix metalloproteinase 2 in the invasion of trophoblasts during early pregnancy, and YY1 deficiency may be involved in the development of RM (25). YY1 regulates cancer cell immune resistance by modulating PD-L1 expression (22). However, the role of YY1 on PD-L1 modulation on trophoblasts has not yet been illustrated.

In this study, we found that patients with RM have reduced levels of YY1 and PD-L1 in their villi compared with the women with normal pregnancy (NP), which was in accordance with the findings that knockdown of *YY1* decreases the expression of PD-L1 (both membrane and soluble forms), eventually decreasing Treg differentiation. Our *in vitro* data found that YY1 directly binds to the transcription start site of *CD274* and promotes its transcription. Apart from the reduced Treg cell pool, the polarization of decidual macrophages (DMs) to the M1 phenotype was also observed in patients with RM (15). Furthermore, we also found increased serum nitric oxide (NO) from women with RM. Interestingly, S-nitroso-N-acetyl-DL-penicillamine (SNAP), the NO donor, decreased the expression of YY1 and PD-L1 in trophoblasts, indicating that increased DMs with the M1 phenotype inhibit YY1/PD-L1 expression *via* producing NO. These results were further confirmed in the SNAP and M1 mouse macrophage-treated healthy pregnant mice. Therefore, our study suggested that increased NO derived from M1 macrophages inhibited YY1/PD-L1 expression in trophoblasts, subsequently resulting in decreased Treg differentiation and the development of RM.

## Materials and methods

### Ethics statement

Our study was approved by the Clinical Trial Ethics Committee and the Animal Research Ethics Committee of Nanjing Drum Tower Hospital, The Affiliated Hospital of Nanjing University Medical School (No. 2022-472-03). Each study subject has signed the informed consent prior to her entrance in the study. All the participants were recruited from the Center for Reproductive Medicine and Obstetrics and Gynecology, Nanjing Drum Tower Hospital, The Affiliated Hospital of Nanjing University Medical School.

### Samples for human villus, decidual tissues, and peripheral blood

There were 17 women with NP and 11 women with RM enrolled in this study. All the volunteers with an early NP had an elective termination at 6–10 weeks of gestation. The gestational age was determined based on the last menstrual cycle or the results of ultrasound examination. The inclusion criteria used for RM patients were as follows: gestation ages were between 6 and 10 weeks, and the women had experienced two or more recurrent pregnancy losses before 20 weeks (15). The basic characteristics of the participants are summarized in the **Supplementary Table 1**. Villous, decidual tissues and peripheral blood were immediately collected, and cell isolation was processed within 2 h. Part of decidual tissues and placental villous were kept at −80°C for cryopreservation until use. Part of these tissues were kept in 10% formalin for fixation.

### Human immune cell isolation

The isolation of decidual immune cells (DICs) was performed as previously described (19). Peripheral naïve CD4^+^ T cells were isolated by magnetic affinity cell sorting using a human naïve CD4^+^ T-cell isolation kit (STEMCELL, Vancouver, BC, Canada).

### Flow cytometry

Human DICs (1×10^6^ cells) and differentiated Treg cells (5×10^5^ cells) after culture were incubated with anti-human CD4-fluorescein isothiocyanate (FITC) antibody (BioLegend, San Diego, CA, USA) and anti-human CD25-Phycoerythrin (PE) (BioLegend) for 30 min in 4°C avoiding from light. After incubation, all the cells were washed using phosphate-buffered saline (PBS). The cells were then fixed and permeabilized with the True-Nuclear Transcription Factor Buffer Set (BioLegend) and then stained with anti-human Forkhead box protein P3 (Foxp3)-Alexa Fluor 647 antibody (BioLegend). Human DMs were incubated with anti-human CD14-FITC antibody (BioLegend), anti-human CD206-PE (BioLegend), and anti-human CD86-Allophycocyanin (APC) (BioLegend) for 30 min in 4°C out of light. For mouse cells, 1×10^6^ cells from uterine were incubated with anti-mouse CD4-FITC antibody (BioLegend), anti-mouse CD25-PE antibody (BioLegend), and anti-mouse Foxp3-APC antibody (BioLegend) for 30 min in 4°C avoiding light. Then, all the cells were washed using PBS. Finally, the cells were resuspended in staining buffer for flow cytometric analysis. All the antibodies were used at 1:20 dilution. Flow cytometry staining buffer, a buffered saline solution containing fetal bovine serum and sodium azide (0.09%), was used to minimize non-specific binding of antibodies. Isotype controls were also used in our study to eliminate background staining caused by non-specific binding of antibodies to cell surfaces. These isotype antibodies include FITC mouse IgG1, κ isotype control antibody, PE mouse IgG1, κ isotype control antibody, Alexa Fluor 647 mouse IgG1, κ isotype control antibody, FITC rat IgG2b, κ isotype control antibody, and PE rat IgG2b, κ isotype control antibody (all from BioLegend). All the data were acquired using a C6+ Flow Cytometer (BD Biosciences, San Jose, CA, USA) and Aria II Flow Cytometer, and analyzed using FlowJo (Tree Star, Ashland, OR, USA).

### Quantitative real-time PCR

The extraction of total ribonucleic acid (RNA) was performed using the TRIzol Reagent (Invitrogen, Carlsbad, CA, USA). Complementary deoxyribonucleic acid (cDNA) synthesis was processed with the same amount of RNA (1 μg). The primers used in this study are presented in **Supplemental Table II**. cDNA (2 μg) was then subjected to qRT-PCR analysis with SYBR Green PCR mix (Vazyme, Nanjing, China). Relative expression was computed by 2^-ΔΔCT^ against *glyceraldehyde-3-phosphate dehydrogenase* (*GAPDH)*.

### Western blot

Protein extraction from human villous, mouse placenta, HTR-8/SVneo cells, and Bewo cells was performed using Cell Lysis Buffer (Cell Signaling, MA, USA), and the total protein concentration was determined by the Pierce BCA Assay Kit (Thermo Fisher, Rockford, IL, USA). An equivalent amount of total protein was boiled for 5 min. After SDS/PAGE, proteins were transferred to the PVDF membrane (Millipore, MA, USA). Membranes were blocked with 5% non-fat milk for 1 h at room temperature. Then, the membrane was incubated with the appropriate primary rabbit anti-YY1 antibodies (Abcam, Cambridge, UK) (1:500) and rabbit anti-PD-L1 antibodies (1:500) (Abcam) at 4°C overnight following blocking. After washing, the membranes were incubated with HRP-conjugated secondary antibody for 2 h at room temperature. With ECL reagents, relative protein blots were visualized. GAPDH (Bioworld, MN, USA) and β-actin (Bioworld) were detected as the loading control.

### Immunohistochemistry

The IHC staining was performed as previously described (26). Human villous and mouse placenta tissue were labeled with rabbit anti-YY1 antibodies (Abcam) (1:500) and rabbit anti-PD-L1 antibodies (Abcam) (1:500), and human decidual tissues were labeled with rabbit anti-inducible nitric oxide synthase (iNOS) antibodies (Bioss, Beijing, China) (1:250). Images were acquired and analyzed with a microscope (Leica, Germany).

### Chromatin immunoprecipitation

A CHIP assay for YY1 was performed using the CHIP kit (Millipore, MA, USA) with a YY1 primary antibody (Abcam). qRT-PCR for the eluted DNA fragments was analyzed as previously described (27).

### Analysis of sPD-L1 in serum by enzyme-linked immunosorbent assay

Peripheral blood samples (2 ml) were collected from each woman into the tube with inert separating gel and coagulant, and serum enrichment was performed by centrifugation for 10 min at 2,000g. The sPD-L1 in human serum was measured using the Human PD-L1 SimpleStep ELISA Kit (Abcam, Cambridge, UK), according to the manufacturer’s protocol.

### NO serum level measurement

NO levels were determined by spectrophotometry using the Griess reagent system (Griess method, WI, USA), according to the manufacturer’s protocol.

### Knockdown of YY1 in HTR-8 cells and Bewo cells

HTR-8/Svneo, a human immortalized extravillous trophoblast (EVT) cell line, was maintained in Roswell Park Memorial Institute (RPMI) 1640 media supplemented with 10% heat-inactivated fetal bovine serum (FBS) and antibiotic–antimycotic cocktail penicillin (100 u/ml) and streptomycin (100 μg/ml) at 37°C in a humidified chamber under normoxic conditions containing 20% O_2_ and 5% CO_2_. Knockdown of YY1 was performed using a specific small interfering RNA (siYY1). The target sequence of siYY1 was CCTGAAATCTCACATCTTA. The oligonucleotides were purchased from RiboBio (Guangzhou, China). The HTR-8 cells were transfected with siYY1 (20 nmol/l) using Lipofectamine 3000 Transfection Reagent (Thermo Fisher, MA, USA). siYY1 transfections were also processed in Bewo cells, a choriocarcinoma cell line, which were cultured in Ham’s F-12K media supplemented with 10% heat-inactivated FBS and antibiotic–antimycotic cocktail penicillin (100 u/ml) and streptomycin (100 μg/ml).

### Preparation of trophoblast conditioned media

To obtain trophoblast conditioned media (TCM), HTR-8/SVneo cells were plated at 5×10^5^ cells/100-mm dish with RPMI 1640 media containing 10% FBS and allowed to attach overnight. Media were then changed to RPMI 1640 media without FBS and incubated with siYY1 or siCtrl. After 5 h, media were changed to RPMI 1640 media containing 10% FBS and incubated up to 48 h. To detect the modulation of NO on trophoblasts, HTR-8/SVneo cells were incubated in RPMI 1640 media with 5% FBS in the presence of S-nitroso-N-acetyl-DL-penicillamine (SNAP) (NO donor) (1 mM) for 48 h. The cell supernatant was collected, clarified, aliquoted, and stored at −80°C until use.

### Treg differentiation

Freshly isolated naïve CD4^+^ T cells were seeded at a density of 5×10^4^ cells per well in 96-well plates and then cultured in complete RPMI 1640 medium (Gibco, Brazil). The purity of sorted cells was generally 90%, as shown in **Supplemental Figure S1**. All the cells were activated by a human CD3/CD28 activator (5 μg/ml) for 3 days with recombinant human interleukin 2 (IL-2) (0.5 U/ml) (STEMCELL, BC, Canada). After 3 days of activation, the cells were incubated with fresh complete RPMI 1640 medium for 2 days. For Treg differentiation, the cells were incubated with TCM in the presence or absence of anti-PD-L1 mAb (eBioscience, CA, USA) (10 ng/ml) for 2 days. At day 7 of the study, cells were collected and assessed for Treg by flow cytometry (BD Biosciences).

### Mice

Eight-week old CBA/J female (n=12), BALB/c male (n=9), and DBA/2 male (n=3) mice were purchased from Huafukang (Beijing, China). Animal use was approved by the Institutional Animal Care and Use Committee of the Nanjing Drum Tower Hospital, The Affiliated Hospital of Nanjing University Medical School, Nanjing, China. All the mice were raised in a specific pathogen-free room with free access to food and water, and mating was carried out. The day when a plug became visible was designated as day 0.5 of pregnancy. Normal pregnant mice (CBA/J×BALB/c) (n=9) were established with CBA/J females mating with BALB/c males. These normal pregnant mice were treated with SNAP (n=3) and mouse M1 macrophages (n=3) at doses of 1 mg/kg or 2 × 10^6^ cells on day 4.5, day 6.5, and day 8.5, respectively. CBA/J females were mated to DBA/2 males to establish the abortion-prone pregnant mice (CBA/J×DBA/2) (n=3). Therefore, all the CBA/J pregnant mice were subdivided into four groups: normal pregnant control group CBA/J×BALB/c (n=3), abortion-prone pregnant group CBA/J×DBA/2 (n=3), SNAP-treated CBA/J×BALB/c pregnant group (n=3), and M1 macrophage-treated CBA/J×BALB/c group (n=3). All pregnant mice were sacrificed on day 10.5 of pregnancy.

### Preparation of mouse M1 macrophages

On day 10.5 of gestation, the femurs and tibias of healthy pregnant mice were aseptically removed to extract bone marrow cells that were cultured overnight (>16 h). Erythrocyte lysate was used to remove red blood cells during the extraction process. Suspension cells were cultured with macrophage colony-stimulating factor (50 ng/ml) for 2 days. The medium was changed to purify and amplify the mouse macrophages. The purified cells were further polarized to M1 macrophages in the presence of lipopolysaccharide (10 ng/ml). After 5 days, the cell surface antigen of mouse macrophages (anti-mouse CD45-FITC antibody, anti-mouse F4/80-PE antibody, anti-mouse CD86-APC, and anti-mouse CD206-Percp-Cy5.5) was detected by flow cytometry (BD Biosciences).

### Preparation of mouse uterine immune cells

The placental and fetal tissues were removed from the executed mice and washed in PBS. Uterine immune cells ware isolated and purified according the previous publication (15).

### Mouse fetal absorption rate analysis

The numbers of absorbed embryos and surviving embryos were calculated as previously described (15, 28).

### Statistical analysis

Data statistical analysis was performed using the Statistical Package for Social Science (SPSS) for windows (Version 18.0 software, SPSS Inc., Chicago, IL, USA) and GraphPad Prism software, version 5 (GraphPad, San Diego, CA, USA). Data were analyzed using the Mann–Whitney test between two groups. The differences among multiple groups were analyzed by one-way ANOVA. Each experiment was repeated at least three times. *P* < 0.05 were considered significant.

## Results

### Lower proportion of Treg cells and decreased expression of PD-L1 and YY1 in patients with RM

**Figure 1:**
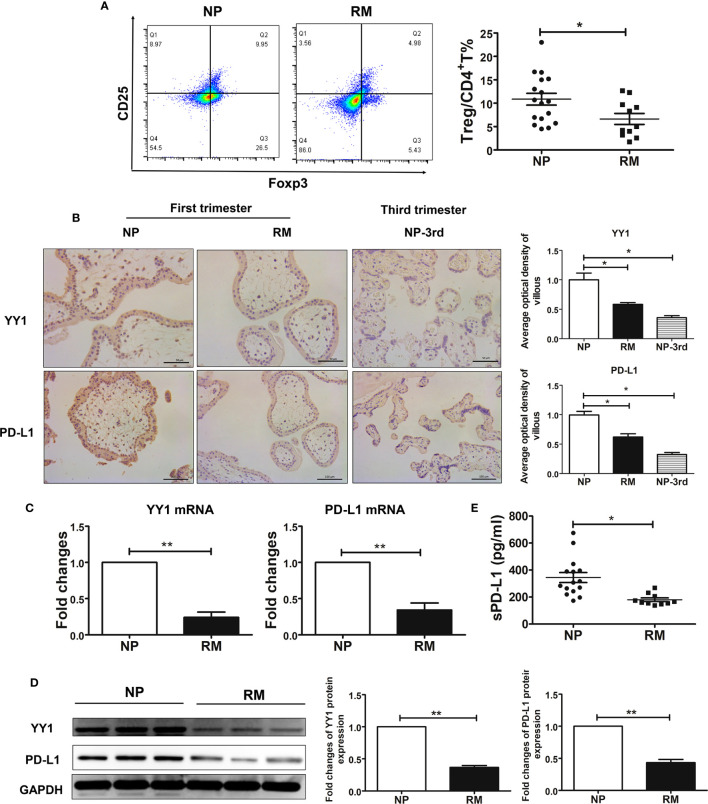
Decreased decidual Treg cell pool and the decreased expression of YY 1 and PD-L1 in trophoblasts from women with normal pregnancy and recurrent miscarriage. **(A)** The proportions of decidual Treg cells (CD4^+^CD25^+^Foxp3^+^) from women with NP (n=17) and RM (n=11) were determined by flow cytometry. **(B)** Staining of YY1 and PD-L1 in maternal villous from women with NP and RM in early pregnancy and third trimester by IHC. **(C, D)** The mRNA levels and protein levels of YY1 and PD-L1 in villous from women with NP (n=6) and RM (n=6). **(E)** The expression of soluble PD-L1 (sPD-L1) in serum from women with NP (n=16) and RM (n=11). Data are presented as the mean± SEM. *P<0.05, **P< 0.01, ***P< 0.001.

Our first objective was to evaluate the Treg population in early-trimester decidual samples from women with NP and RM. In this study, Treg cells were defined as CD4^+^CD25^bright^Foxp3^+^ as many publications had reported (29–35). The flow cytometry strategy is represented in **Supplementary Figure S2**. Compared with women with NP, decreased percentages of Treg cells were observed in patients with RM (**Figure 1A**). To assess the expression of PD-L1 and YY1 in villous tissues obtained from women with NP and RM, we performed IHC staining analysis. It was found that PD-L1 and YY1 were present in trophoblast cells (**Figure 1B**). The intensity values of PD-L1 and YY1 in the villous from RM patients were lower than those from NP. Weak positive signals for YY1 and PD-L1 was also detected in trophoblasts from women with third-trimester placental tissue (**Figure 1B**). The data from Western blot and qRT-PCR also showed that the expressions of PD-L1 and YY1 in villous tissues of RM patients were significantly lower than those of women with NP (**Figures 1C, D**). Next, we evaluated the soluble PD-L1 (sPD-L1) in serum sampled from women with NP and RM using the PD-L1 SimpleStep ELISA kit. As shown in **Figure 1E**, sPD-L1 was detected in serum from pregnant women. However, the serum sPD-L1 was decreased in women with RM. These results indicated that YY1 might be involved in the pathogenesis of RM *via* inhibiting PD-L1 expression.

### YY1 promotes PD-L1 expression in trophoblasts favoring Treg differentiation

**Figure 2:**
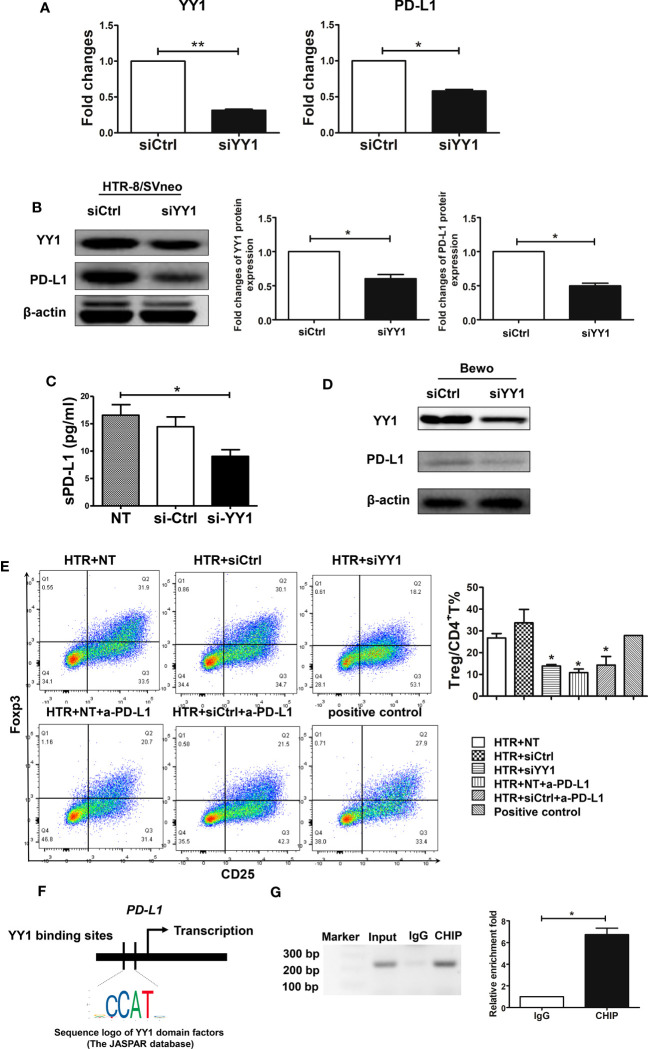
The modulatory effects and mechanism of YY1 on PD-L1 in trophoblasts. **(A, B)** qRT-PCR and Western blot analysis of *YY1* and *PD-L1* expression in HTR-8/SVneo cells transfected with siCtrl and siYY1 (n=3, for each group) after 48 h. **(C)** HTR-8 cells were transfected with one of the abovementioned two siRNA and incubated for 48 h. The TCM was collected, and the secretion of sPD-L1 from trophoblasts was evaluated by the PD-L1 SimpleStep ELISA kit (n=5, for each group). **(D)** Bewo cells were transfected with siCtrl and siYY1 and incubated for 48 h. The expressions of YY1 and PD-L1 were measured by Western blot. **(E)** Peripheral naïve CD4^+^ T cells were isolated from women with NP and cultured with TCM from above for 7 days (n=3, for each group). The percentages of Treg cells (CD4^+^CD25^+^Foxp3^+^) were analyzed by flow cytometry. **(F)** Schematic diagram of YY1 binding location and sequence on the human *CD274* (PD-L1) gene. Identified YY1 binding sequence was compared with consensus sequence (the CCAT sequence is essential for YY1 binding). **(G)** The CHIP assay was carried out in villous from women with NP. Input served as a positive control for CHIP. IgG was used as a negative control for CHIP. Data are presented as the mean± SEM. *P<0.05.

A recent publication has shown that PD-L1 is generally regulated by YY1 (22). As YY1 was expressed in first-trimester trophoblasts, we next investigated whether YY1 is involved in the modulation of PD-L1. We used HTR-8 cells, a first-trimester human trophoblast-derived cell line, which were transfected with siYY1. The transcription and translation levels of *YY1* were decreased after siYY1 transfection in trophoblasts (**Figures 2A, B**). PD-L1 expression also downregulated with YY1 knockdown (**Figures 2A, B**). Next, we determined whether PD-L1 was secreted by collecting culture supernatants form the trophoblast cells after 24 h, and sPD-L1 was measured with the PD-L1 SimpleStep ELISA kit. As shown in **Figure 2C**, sPD-L1 was detected in the supernatants of HTR-8 cells, suggesting constitutive secretion of sPD-L1 by trophoblast cells. However, the production of sPD-L1 was decreased in the presence of siYY1 (**Figure 2C**), further indicating that YY1 modulates both membrane and soluble PD-L1 in trophoblasts. The modulatory effects of YY1 on PD-L1 were further confirmed with Bewo cells. Compared with the control group, siYY1 transfection inhibited the expression of YY1 and PD-L1 in Bewo cells (**Figure 2D**).

PD-L1 was constitutively expressed by trophoblast cells and has the capacity to modulate decidual immune cells, such as Treg cells (36). To further confirm the role of YY1 on PD-L1 modulation, naïve CD4^+^ T cells were incubated with TCM after siYY1 transfection in the presence or absence of a specific PD-L1-blocking antibody (anti-PD-L1 mAb), which consequently blocks the activation of the PD-1/PD-L1 pathway. The flow cytometry strategy for Treg differentiation is represented in **Supplementary Figure S3**. We found that TCM promoted Treg differentiation (**Figure 2E**). In contrast, blocking the engagement of PD-1 and PD-L1 decreased Treg differentiation. The conditioned media from siYY1-transfected trophoblasts also downregulated Treg differentiation indicating the regulation of YY1 on PD-L1 expression (**Figure 2E**).

To clarify the possible mechanism of YY1 regulating PD-L1, we predicated the binding site of YY1 on the *CD274* (PD-L1) gene through Jaspar Software (**Figure 2F**). In addition, CHIP analysis of human early villous showed that endogenous YY1 occupied the promoter region of the *CD274* gene (**Figure 2G**). These results indicate that YY1 is a transcriptional activator of *PD-L1*.

### SNAP mimic NO inhibits YY1/PD-L1 expression and Treg differentiation

**Figure 3:**
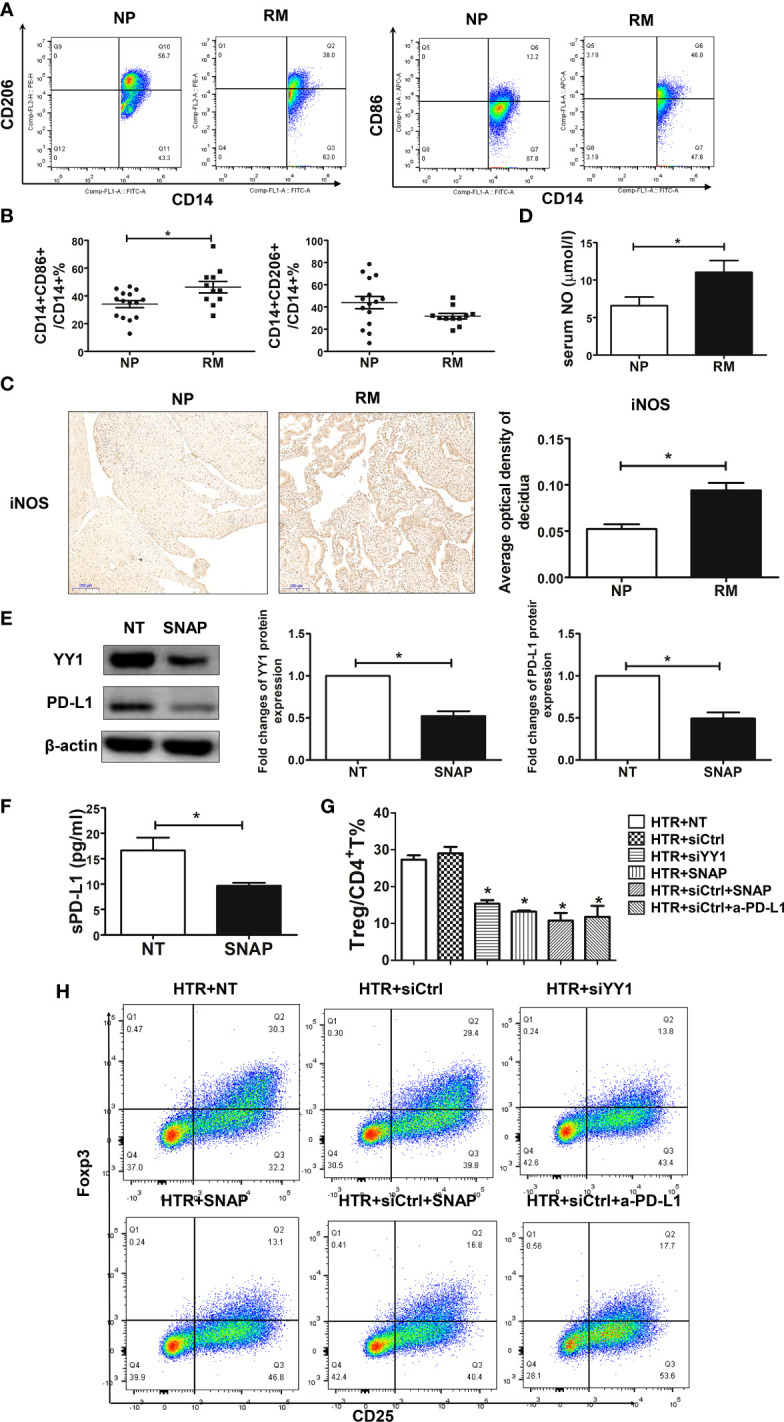
SNAP downregulated the expression of YY1 and PD-L1 in trophoblasts leading to decreased Treg differentiation. **(A, B)** The percentages of M1 (CD14^+^CD86^+^) and M2 (CD14^+^CD206^+^) from women with NP (n=15) and RM (n=11) were determined by flow cytometry. **(C)** Single staining of iNOS in maternal decidua from women with NP and RM were analyzed by IHC (n=3, for each group). **(D)** The production of NO in serum from women with NP (n=13) and RM (n=11) were analyzed by the Griess reagent system. **(E)** HTR-8 cells were exposed to SNAP (NO donor) (1 mM) for 48 h, and the expressions of YY1 and PD-L1 were determined by Western blot (n=3, for each group). **(F)** The production of sPD-L1 from HTR-8 cells with or without SNAP (n=4, for each group) was evaluated by the PD-L1 SimpleStep ELISA kit. **(G, H)** Peripheral naïve CD4^+^ T cells were isolated from women with NP and treated with TCM from siCtrl-, siYY1-, and SNAP-treated HTR-8 cells in the presence or absence of a-PD-L1 mAbs (n=3, for each group). The percentages of Treg cells (CD4^+^CD25^+^Foxp3^+^) were analyzed by flow cytometry. Data are the mean± SEM. *P<0.05.

During pregnancy, placental trophoblasts closely communicated with decidual immune cells. Placental trophoblasts modulate Treg differentiation *via* PD-L1. Conversely, trophoblasts were also regulated by decidual immune cells. Therefore, we hypothesized that the decreased expression of PD-L1 observed in the villous from patients with RM might be associated with increased NO produced from DMs. To test the hypothesis, we first evaluated the profile of DMs in decidual samples from women with NP and RM. As previously, M1 macrophages were defined as CD14^+^CD86^+^ and M2 macrophages were defined as CD14^+^CD206^+^ (15). The flow cytometry strategy for DMs is represented in **Supplementary Figure S4**. Both M1 and M2 DMs were identified in women with NP, and M2 was the dominant phenotype, as demonstrated by the presence of higher percentages of CD14^+^CD206^+^ (**Figure 3A**). We also observed the two populations of DMs in women with RM, although the dominant phenotype of DMs in these patients was M1, namely, higher percentage of M1 and lower percentage of M2 (**Figure 3B**). We then measured the expression of iNOS in the decidua sampled from women with NP and RM and observed that the expression of iNOS was higher in decidua from RM (**Figure 3C**). These findings indicated higher production of NO from M1 macrophages in patients with RM. To validate whether the increased M1 macrophages and iNOS expression in RM contributed to more NO production, we determined NO levels in serum using the Griess reaction. Our data showed that in patients with RM, serum NO levels were higher compared with the women with NP (**Figure 3D**).

To further investigate the role of NO in regulating PD-L1 expression in villous, we exposed HTR-8 cells to S-nitroso-N-acetyl-DL-penicillamine (SNAP) (1 mM) for 24 h. SNAP is the NO donor and acts as a regulatory molecule in the reversal of immune resistance, by repressing SNAIL, YY1, the pro-survival NF-κB pathway, and the anti-apoptotic AKT pathway (21). We found that the expression of YY1 and PD-L1 was inhibited in the HTR-8 cells in the presence of SNAP (**Figure 3E**). Interestingly, the production of sPD-L1 by HTR-8 cells was also downregulated with SNAP (**Figure 3F**). To further determine the modulatory effects of SNAP-treated trophoblasts on Treg differentiation, naïve CD4^+^ T cells were exposed to the TCM treated with SNAP, siCtrl, and siYY1. Our data showed that Treg differentiation was inhibited in the presence of supernatant from SNAP-treated HTR-8 cells (**Figure 3G**). However, there was no significance on Treg differentiation with the supernatant from SNAP and siYY1-treated HTR-8 cells (**Figure 3G, H**). These data suggest that NO from macrophages inhibits YY1/PD-L1 expression on trophoblasts, further resulting in less Treg differentiation.

### SNAP increased the embryo resorption rate and scaled down the Treg cell pool *via* inhibiting YY1 and PD-L1 in mice

**Figure 4:**
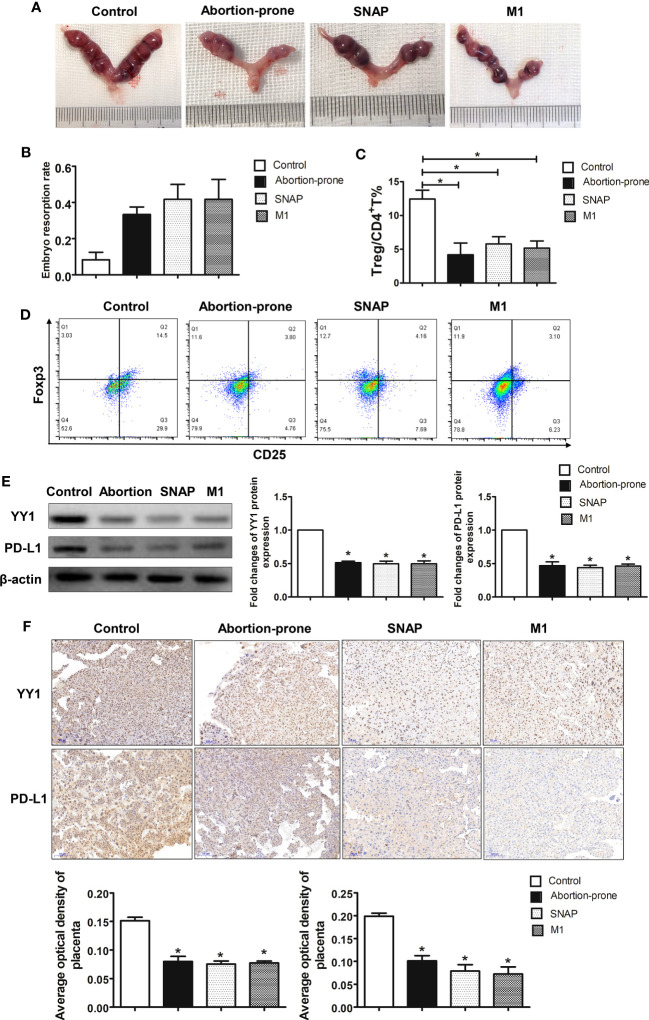
SNAP inhibited YY1/PD-L1 expression on placenta accompanied by decreased decidual Treg population and increased embryo resorption in mice. **(A, B)** The embryo resorption rates of normal pregnant control mice (CBA/J×BALB/c) (n=3), abortion-prone mice (CBA/J×DBA/2) (n=3), SNAP-treated normal pregnant mice (n=3) and mouse M1 macrophage-treated normal pregnant mice (n=3) were analyzed. **(C, D)** The percentages of Treg cells in uterine among the four groups were analyzed by flow cytometry. **(E)** The expressions of YY1 and PD-L1 in placenta among the four groups were measured by Western blot. **(F)** Staining of YY1 and PD-L1 on placenta among the four groups were analyzed by IHC. Data are the mean± SEM. *P<0.05.

To further explore the modulatory role of NO on the expression of YY1 and PD-L1 in early pregnancy, we established an allogenic normal pregnant model by mating CBA/J females with BALB/c males (CBA/J×BALB/c). Then, the normal pregnant mice were challenged with SNAP and mouse M1 macrophages. We also used the CBA/J females mating with DBA/2 males to establish the abortion-prone pregnant model (CBA/J×DBA/2). The increased embryo resorption rate was induced by administration of SNAP and mouse M1 macrophages to CBA/J×BALB/c pregnant mice. A similar rate of fetal loss was also observed in the abortion-prone mice CBA/J×DBA/2 (**Figure 4A, B**).

Next, we further investigated whether fetal loss observed in the CBA/J×BALB/c pregnant mice receiving SNAP and mouse M1 macrophages was due to Treg dysfunction. Thus, the proportion of uterine Treg cells (CD4^+^CD25^+^Foxp3^+^) was evaluated by flow cytometry on day 10.5 sampled from four groups (**Figure 4C**). The flow cytometry strategy for Treg differentiation is represented in **Supplementary Figure S5**. We observed a significant decrease in the proportion of Treg cells in the uterus from the abortion-prone mice (**Figure 4D**). Interestingly, the CBA/J×BALB/c mice treated with SNAP and mouse M1 macrophages also showed lower percentages of Treg cells (**Figure 4D**).

To validate whether the diminished Treg cell pool in the CBA/J×DBA/2 mice was associated with changes on the expression of YY1 and PD-L1, we determined YY1 and PD-L1 levels in placenta by Western blot and IHC. We found that in the CBA/J×DBA/2 mice, the protein levels of YY1 and PD-L1 were significantly lower in placenta compared with the healthy control group (**Figure 4E**). This was the same case for CBA/J×BALB/c mice administrated with SNAP or M1 macrophages, where we found lower YY1 and PD-L1 expressions in placenta (**Figure 4E**). These results were further confirmed by the IHC data, showing lower expressions of YY1 and PD-L1 in the placenta from the abortion-prone CBA/J×DBA/2 mice, SNAP-treated CBA/J×BALB/c mice, and mouse M1 macrophage-treated CBA/J×BALB/c mice (**Figure 4F**). These data further demonstrated that Treg differentiation was associated with YY1/PD-L1 signaling, which was modulated by M1 macrophages derived NO. A higher percentage of M1 macrophages or higher production of NO inhibited the expression of YY1 and PD-L1 on placenta, which may be potentially responsible for the lower proportion of Treg cells and fetal loss in RM.

## Discussion

In this study, we firstly examined the effects of YY1 on the modulation of PD-L1 expression in trophoblasts and Treg differentiation during human early pregnancy. These findings indicated that YY1 has a positive role in promoting PD-L1 expression and Treg immunity found in the human decidua. Knockdown of YY1 diminished PD-L1 expression in trophoblasts and subsequently less Treg differentiation. This might be through YY1 binding directly to the promoter region of *CD274*. Furthermore, we explored the modulatory role of M1 macrophages on the expression of YY1 *via* secreting NO. We observed the presence of the decreased Treg cell pool and a higher percentage of DM with an M1 phenotype in decidua from patients with RM. Compared with NP, higher levels of serum NO were observed in women with RM. Interestingly, our *in vitro* data further confirmed the modulatory capability of NO on the expression of YY1 and PD-L1 in trophoblasts and subsequent Treg differentiation. Either alteration in the profile of DMs or higher amount of NO may result in an increased fetal loss due to the decreased expression of YY1 and PD-L1 in placenta and the diminished Treg cell pool.

Treg cells are essential mediators of pregnancy tolerance. In line with previous studies, we confirmed the presence of decidual Treg cells in women with NP (19). However, a lower percentage of Treg cells was observed in decidua from RM, suggesting that a lower proportion of Treg cells may be an underlying cause for RM (12, 18). Human placenta is emerging as an immune organ. Placenta-derived trophoblasts carrying paternal antigens are in close contact with the maternal immune cells at the maternal–fetal interface. Instead of being passively accepted, trophoblast cells function positively to educated decidual immune cells into tolerant phenotype (37). The former findings indicated that placenta promotes Treg differentiation and normal pregnancy *via* the PD-L1/PD-1 axis (14, 38). However, decreased PD-L1 (especially sPD-L1) in trophoblasts was supposed associated with pregnancy loss (14, 39–41). Our data also showed decreased expression of PD-L1 in both membrane and soluble forms in women with RM. However, the mechanisms underlying PD-L1 regulation are still unknown.

Being a transcriptional modulator, YY1 has been confirmed to participate in various processes, such as cell survival and metabolism. Previous publications demonstrated that YY1 regulates the expression of PD-L1 *via* several indirect mechanisms, including cytokines, P53, and the PTEN/PI3K/AKT/mTOR pathway (29). However, the major mechanism of YY1 regulating PD-L1 is supposed very likely by governing PD-L1 expression (26). In this study, the immortalized primary trophoblast cells, HTR-8/Svneo cell line, were introduced. Although primary cells derived from human tissue are considered a “gold standard” for *in vitro* investigation of cellular physiology, they are logistically challenging to harvest, obtain, and maintain. Primary placental cells have been utilized to investigate placental physiology, but these cells are heterogenous and cannot be used in a high-throughput manner (42). The choriocarcinoma cell line, Bewo, JAR, and JEG-3, are highly malignant, contain abnormal numbers of chromosomes, have been passaged through the hamster cheek pouch for several years, and have a substantially different transcriptomic profile from EVT (43–46). The placental cell line and tissue proteomes are vastly different, but Bewo and JEG-3 cells showed greater resemblance to the tissue in the expression of xenobiotic and steroid disposition proteins (42). The first evCTB cell line was developed by Graham et al. (47) and named HTR-8/SVneo. It was generated using freshly isolated evCTB from first-trimester placenta and transfected with a plasmid containing the simian virus 40 large T antigen (SV40). In comparison with BeWo, JEG-3, and JAR, the HTR-8/SVneo cell line contains a heterogeneous population of trophoblast mesenchymal cells (48). HTR-8 monolayer cells grown on plastic did not stain for human leukocyte antigen G (HLA-G). On the other hand, spheroid-derived cells cultured overnight on plastic show HLA-G protein expression (49). Therefore, whether these are truly representative of normal trophoblast is controversial. A major advancement in the trophoblast field has been the establishment of trophoblast organoids that can differentiate from villous into the two main trophoblast cell lineages: syncytiotrophoblast and EVT (50, 51). These will clearly be an important tool for studying trophoblast invasion as they vigorously invade in 3D into Matrigel. In addition to organoids, human trophoblast stem cells have also been derived from blastocysts and first-trimester placentas. These cultures are in 2D but can also be differentiated to produce EVT (52). The development of both 2D and 3D human trophoblast stem cells is a paradigm shift in the tools used to study trophoblast invasion. Above all, the HTR-8/SVneo cell line meets the requirement of experiments and has strong feasibility for two reasons: (i) We aimed to explore the modulation of trophoblast on T-cell differentiation rather than trophoblast invasion, and (ii) trophoblast-derived PD-L1 might induce Treg differentiation instead of HLA-G and steroid hormone (such as progesterone) (53–55), which had been shown effective on promoting Treg differentiation. We observed that knockdown of YY1 on trophoblast is accompanied by decreased expression of membranes PD-L1 and sPD-L1, which subsequently results in less Treg differentiation. Furthermore, we also proved that YY1 could modulate *CD274* transcription *via* directly binding to its promoter region. However, the potential mechanism underlying YY1 modulation is unclear.

As we have mentioned above, trophoblasts were closely associated with immune cells. Spatially, DMs were located in proximity to invasive trophoblasts (56). Therefore, the expression of YY1 in trophoblasts might be regulated by DMs. In line with former findings, RM is featured with a higher percentage of DMs with the M1 phenotype (22). Accordingly, a higher level of iNOS was observed in decidua from women with RM, which was in line with former findings (57). Therefore, higher production of NO from M1 macrophages might inhibit YY1 expression (21). As expected, higher serum NO was observed in women with RM. Interestingly, our *in vitro* data confirmed that SNAP, NO donor, administration diminished YY1 expression, which further results in lower production of both membrane and soluble forms of PD-L1. Subsequently, decreased sPD-L1 was associated with less differentiation of Treg cells from naïve CD4^+^T cells.

To further test whether DM-derived NO has influences on the YY1/PD-L1 axis and pregnancy, we used an animal model where we found that a higher level of NO inhibited the expression of YY1 and PD-L1 in placenta, which was associated with increased fetal resorption and decreased Treg cell pool. Similarly, Aisemberg et al. showed that administration of NO to pregnant mice promoted fetal rejection (58). Interestingly, YY1 and PD-L1 expression in mouse placenta and Treg cell pool were diminished in the abortion-prone mice and administration of SNAP and mouse M1 macrophages to healthy pregnant mice increased fetal loss. Although still controversial, we also observed a similar pregnant outcome and the expression of YY1 and PD-L1 between the SNAP and M1 mouse macrophage-treated pregnant mice. Thus, higher M1 macrophage-derived NO might result in higher fetal loss by diminishing YY1 and PD-L1 expression in trophoblasts, which results in less differentiation of Treg cells.

**Figure 5:**
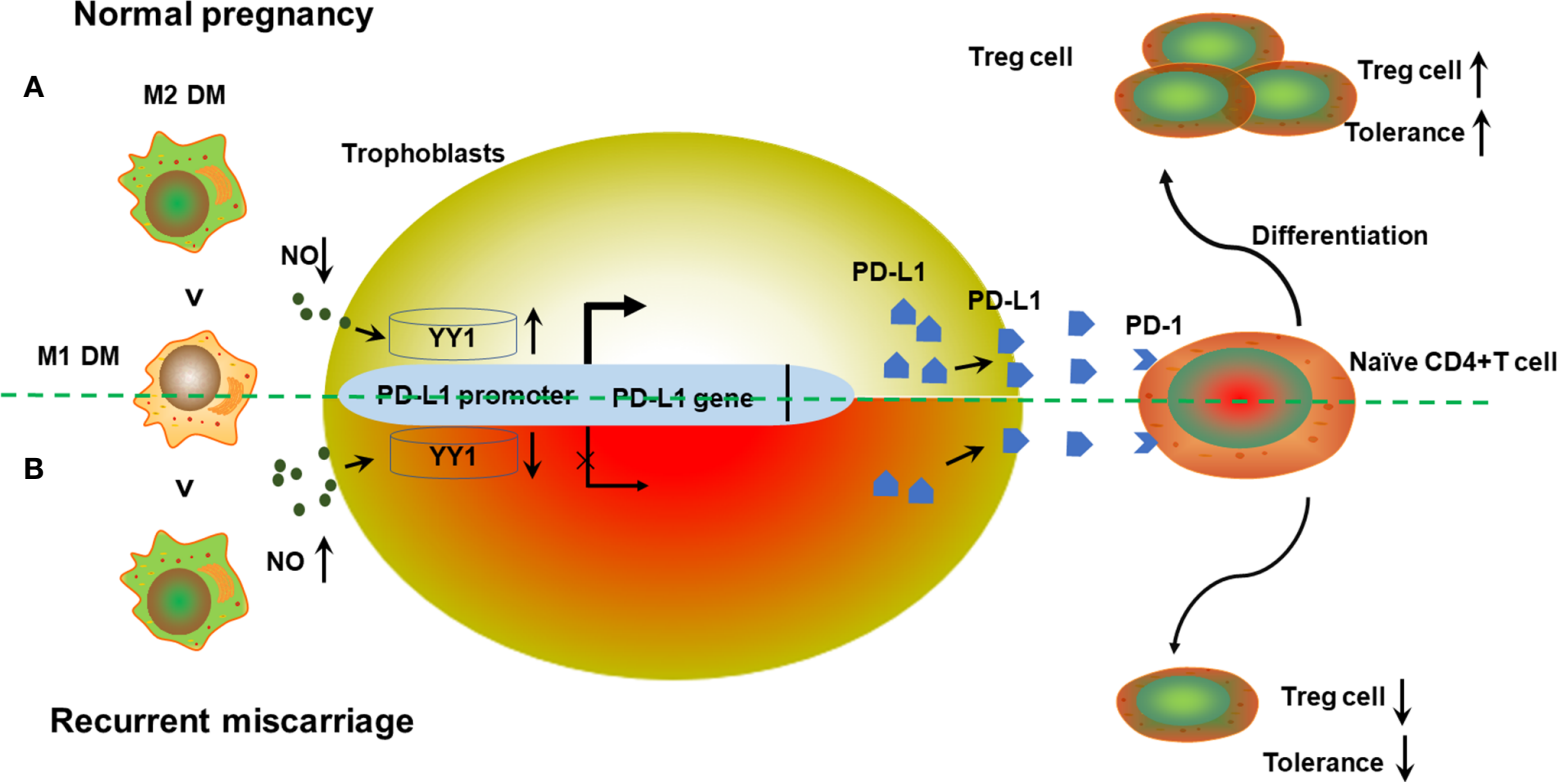
Schematic diagram of the action of M1 macrophage derived NO on trophoblasts’ YY1/PD-L1 expression at the maternal–fetal interface to maintain normal pregnancy. **(A)** During early pregnancy, DMs show the predominant M2 phenotype after implantation. A relative lower production of NO from M1 macrophages promotes YY1 expression in trophoblasts. Increased expression of YY1 enhances both membrane and soluble PD-L1 *via* binding to the transcription site of *CD274*. Higher secretion of PD-L1 further promotes Treg differentiation *via* engagement with PD-1 on T cells, contributing to Treg differentiation and normal pregnancy. **(B)** However, the predominance of M1 macrophages in decidua is accompanied by higher production of NO, which further inhibits the expression of YY1 and PD-L1. Accordingly, the Treg cell pool was reprogrammed, with less Treg differentiation. Decreased Treg population results in the development of RM with compromised maternal tolerance.

Although we still could not conclude whether fetal loss was the cause or consequence of the decreased expression of YY1 and PD-L1 in trophoblasts in women with RM, we demonstrated that NO originated from M1 macrophages is critical for YY1/PD-L1 expression, which is necessary for the success of pregnancy (**Figure 5**). The predominance of the M1 phenotype for DMs might be responsible for the decreased expression of YY1 and PD-L1 and subsequent lower proportion of Treg cells observed at the maternal–fetal interface in RM *via* producing NO. These results provide a new perspective for understanding the mechanisms that promote maternal–fetal tolerance. Our research also indicates that targeting macrophage polarization may be another new therapeutic strategy to prevent pregnancy failure.

## Data availability statement

The original contributions presented in the study are included in the article/**Supplementary Material**, further inquiries can be directed to the first author (Email: yhzhangls@163.com).

## Ethics statement

The studies involving human participants were reviewed and approved by Clinical Trial Ethics Committee and the Animal Research Ethics Committee of Nanjing Drum Tower Hospital, The Affiliated Hospital of Nanjing University Medical School. The patients/participants provided their written informed consent to participate in this study. The animal study was reviewed and approved by Clinical Trial Ethics Committee and the Animal Research Ethics Committee of Nanjing Drum Tower Hospital, The Affiliated Hospital of Nanjing University Medical School.

## Author contributions

YZ conceived and carried out experiments and drafted the manuscript. PQ and JJ carried out part of the experiment and data analysis. HW and XW coordinated the sample collection and data analysis. JM and HS revised the manuscript. All authors reviewed the manuscript. All authors contributed to the article and approved the submitted version

## Supplementary material

Supplementary Figure 1 Gating strategy for determination of naïve CD4+T cells in peripheral by flow cytometry. **(A)** Lymphocytes were measured against forward and side scatter parameters. **(B)** Cells were further displayed on a plot of CD4 expression. The purification of CD4^+^T cells is generally 90% (96.3%*93.7%=90.2%).

Supplementary Figure 2 Gating strategy for determination of decidual Treg cells from women with NP and RM. **(A)** Lymphocytes were measured against forward and side scatter parameters. **(B)** Cells were further displayed on a plot of CD4 expression (CD4^+^ gate). **(C)** Cells contained within CD4^+^ gate were further displayed on a plot of CD25 versus Foxp3 expression.

Supplementary Figure 3 Gating strategy for determination of differentiated Treg cells from naïve CD4^+^T cells. **(A)** Lymphocytes were measured against forward and side scatter parameters. **(B)** Cells were further measured with LIVE/DEAD Fixable Blue stain fluorescence. **(C)** Live cells were further displayed on a plot of CD4 expression (CD4^+^ gate). **(D)** Cells contained within CD4^+^ gate were further displayed on a plot of CD25 versus Foxp3 expression.

Supplementary Figure 4 Gating strategy for determination of DMs from women with NP and RM. **(A)** Macrophages were measured against forward and side scatter parameters. **(B)** Cells were further displayed on a plot of CD14 expression (CD14^+^ gate). **(C)** Cells contained within CD14^+^ gate were further displayed on a plot of CD86 versus CD14 expression or a plot of CD206 versus CD14xpression.

Supplementary Figure 5 Gating strategy for determination of uterine Treg cells in mice. **(A)** Lymphocytes were measured against forward and side scatter parameters. **(B)** Cells were further displayed on a plot of CD4 expression (CD4^+^ gate). **(C)** Cells contained within CD4^+^ gate were further displayed on a plot of CD25 versus Foxp3 expression.
