# Supplementary figures and images for: Decidual macrophages derived NO downregulates PD-L1 in trophoblasts leading to decreased Treg cells in recurrent miscarriage
Source: Front Immunol. 2023 Jul 14;14:1180154. doi: 10.3389/fimmu.2023.1180154 (PMC10379637; doi:10.3389/fimmu.2023.1180154)

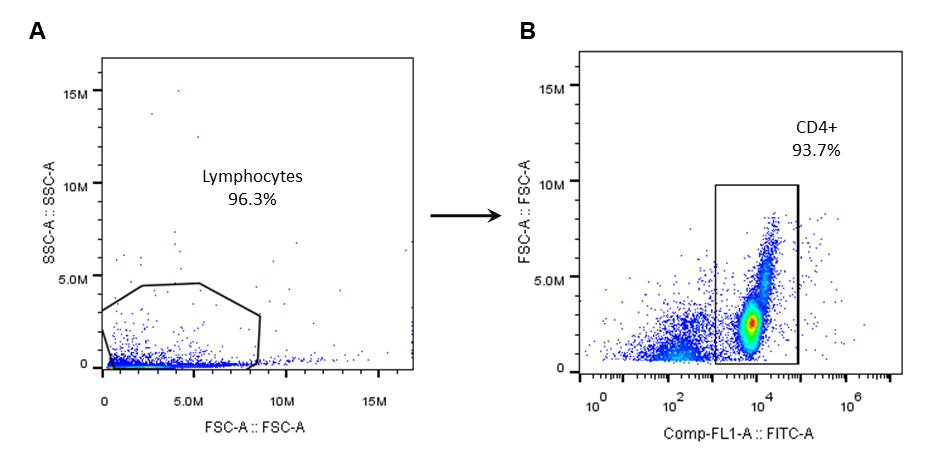

Supplement: Supplementary Figure 1 — Gating strategy for determination of naïve CD4+T cells in peripheral by flow cytometry. (A) Lymphocytes were measured against forward and side scatter parameters. (B) Cells were further displayed on a plot of CD4 expression. The purification of CD4+T cells is generally 90% (96.3%*93.7%=90.2%). [file Image_1.tif]

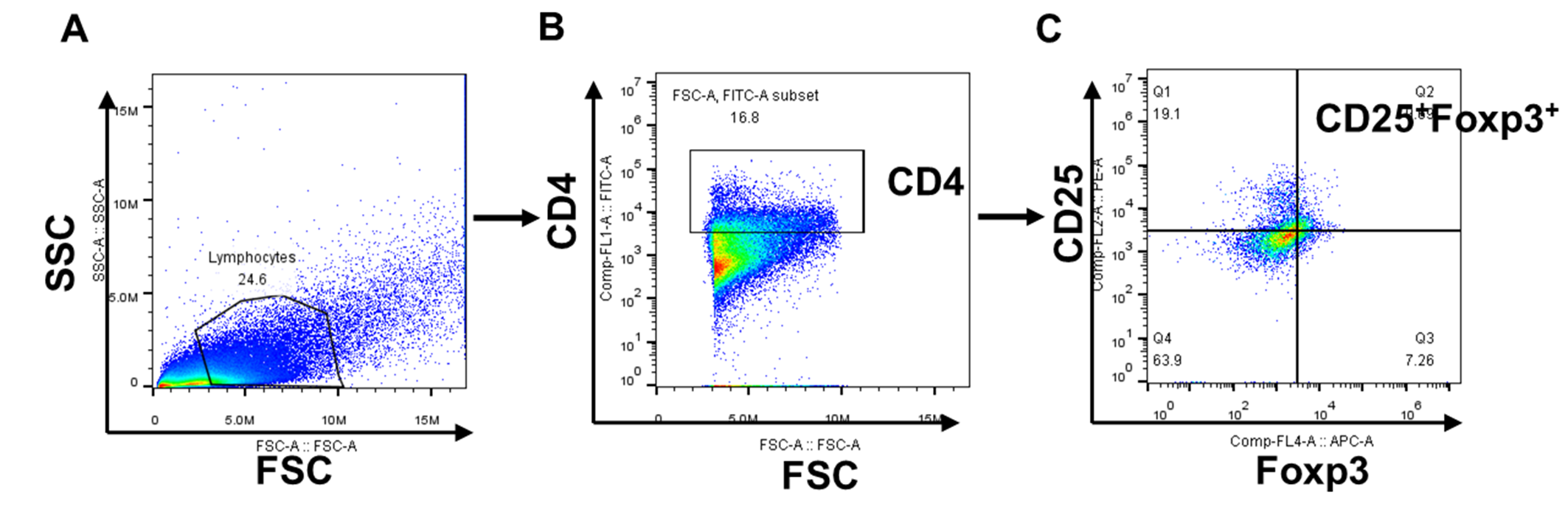

Supplement: Supplementary Figure 2 — Gating strategy for determination of decidual Treg cells from women with NP and RM. (A) Lymphocytes were measured against forward and side scatter parameters. (B) Cells were further displayed on a plot of CD4 expression (CD4+ gate). (C) Cells contained within CD4+ gate were further displayed on a plot of CD25 versus Foxp3 expression. [file Image_2.tif]

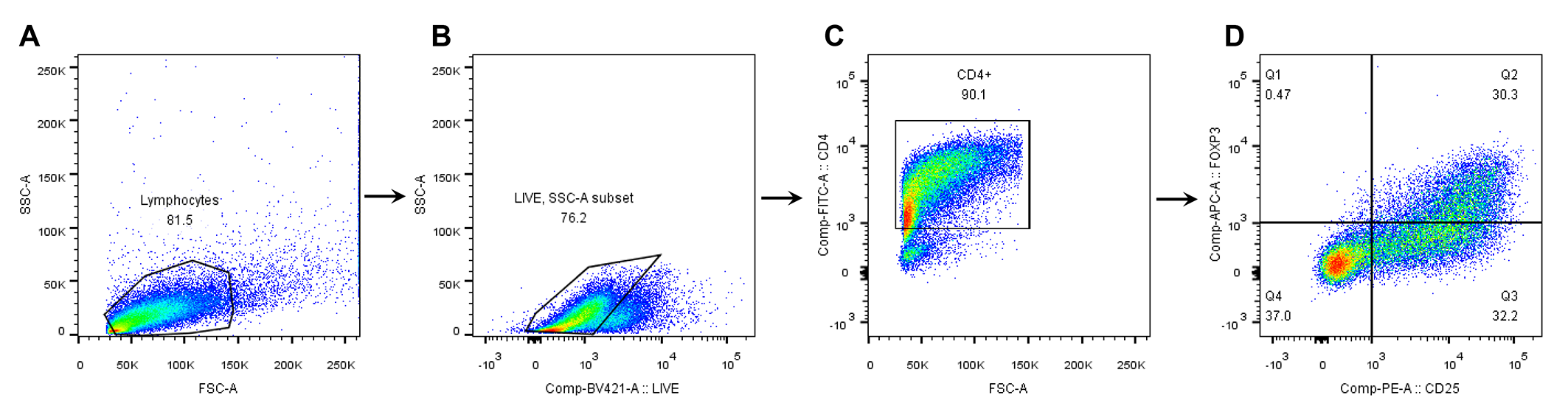

Supplement: Supplementary Figure 3 — Gating strategy for determination of differentiated Treg cells from naïve CD4+T cells. (A) Lymphocytes were measured against forward and side scatter parameters. (B) Cells were further measured with LIVE/DEAD Fixable Blue stain fluorescence. (C) Live cells were further displayed on a plot of CD4 expression (CD4+ gate). (D) Cells contained within CD4+ gate were further displayed on a plot of CD25 versus Foxp3 expression. [file Image_3.tif]

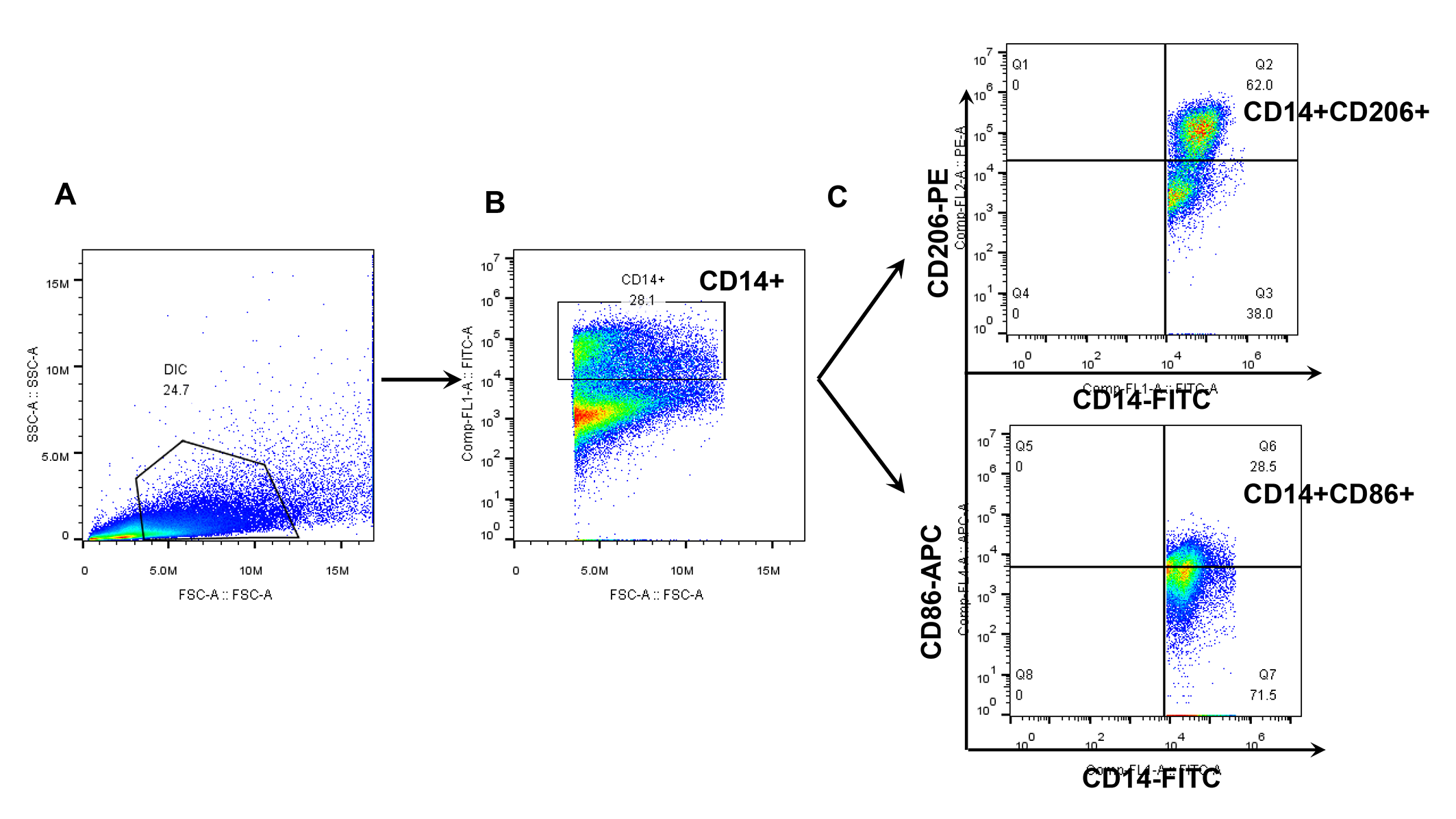

Supplement: Supplementary Figure 4 — Gating strategy for determination of DMs from women with NP and RM. (A) Macrophages were measured against forward and side scatter parameters. (B) Cells were further displayed on a plot of CD14 expression (CD14+ gate). (C) Cells contained within CD14+ gate were further displayed on a plot of CD86 versus CD14 expression or a plot of CD206 versus CD14xpression. [file Image_4.tif]

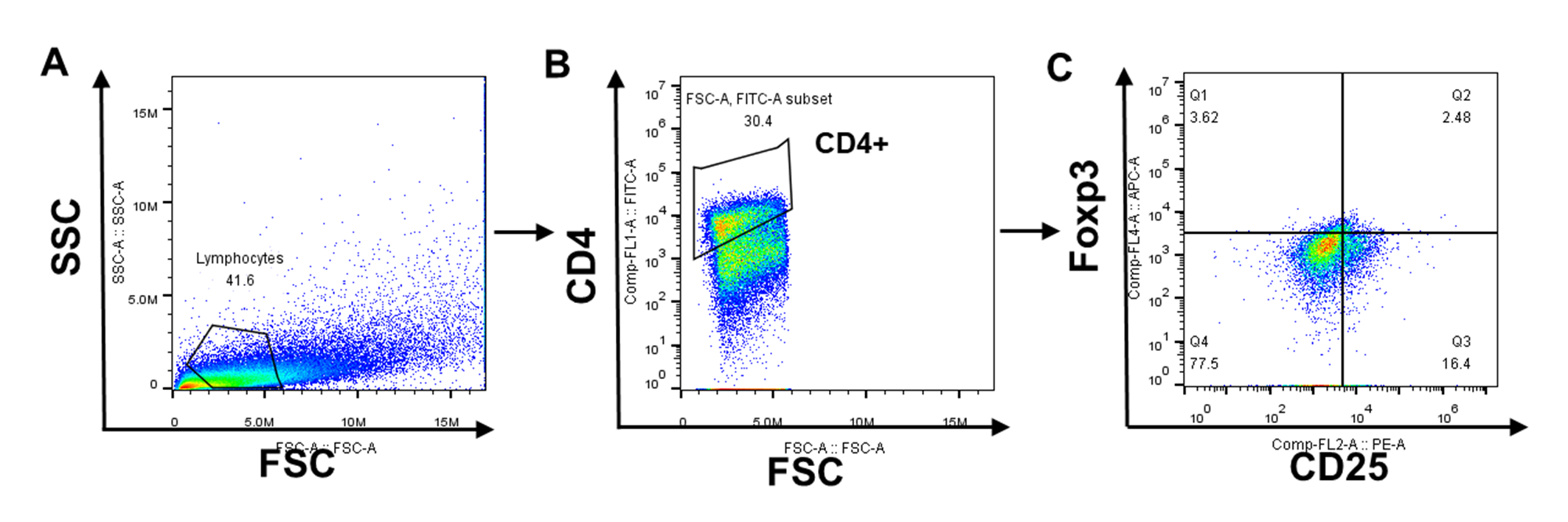

Supplement: Supplementary Figure 5 — Gating strategy for determination of uterine Treg cells in mice. (A) Lymphocytes were measured against forward and side scatter parameters. (B) Cells were further displayed on a plot of CD4 expression (CD4+ gate). (C) Cells contained within CD4+ gate were further displayed on a plot of CD25 versus Foxp3 expression. [file Image_5.tif]
