# Supplementary material for: Decidual macrophages derived NO downregulates PD-L1 in trophoblasts leading to decreased Treg cells in recurrent miscarriage
Source: Front Immunol. 2023 Jul 14;14:1180154. doi: 10.3389/fimmu.2023.1180154 (PMC10379637; doi:10.3389/fimmu.2023.1180154)
Supplement: Supplementary file 6 [file Table_1.docx]

**Supplementary Table 1** Characteristics of the women with NP and RM

|  | NP (n=17) | RM (n=11) |
| --- | --- | --- |
| Age (years) | 29.05+4.95 | 27.21+3.87 |
| Gestational age (weeks) | 7.16+1.01 | 8.67+1.85 |
| Gravidity | 1.35+1.11 | 2.35+0.47 |
| Parity | 0.70+0.64 | 0+0 |
| Abortion for elective termination | 0.65+0.65 | / |
| Miscarriages | / | 2.35+0.47 |

Gravidity=number of pregnancies; Parity=the number of births carried to a viability; Miscarriages=number of pregnancies which ended in miscarriage or abortion; Data are analyzed by *t* test and shown in mean+SEM.
