# Supplementary material for: Decidual macrophages derived NO downregulates PD-L1 in trophoblasts leading to decreased Treg cells in recurrent miscarriage
Source: Front Immunol. 2023 Jul 14;14:1180154. doi: 10.3389/fimmu.2023.1180154 (PMC10379637; doi:10.3389/fimmu.2023.1180154)
Supplement: Supplementary file 7 [file Table_2.docx]

**Supplementary Table Ⅱ** Primer sequences used in quantitative RT-PCR experiments.

| Gene ID | Sense primer (5’-3’) | Antisense primer (5’-3’) |
| --- | --- | --- |
| ACTB | AGATGACCCAGATCATGTTTGAG | AGGTCCAGACGCAGGATG |
| PD-L1 | GCTTTTCAATGTGACCAGCA | TGGCTCCCAGAATTACCAAG |
| YY1 | AGAATAAGAAGTGGGAGCAGAAGC | ACGAGGTGAGTTCTCTCCAATGAT |
